# Addressing the complex phylogenetic relationship of the Gempylidae fishes using mitogenome data

**DOI:** 10.1002/ece3.10217

**Published:** 2023-06-21

**Authors:** Siphesihle Mthethwa, Aletta E. Bester‐van der Merwe, Rouvay Roodt‐Wilding

**Affiliations:** ^1^ Molecular Breeding and Biodiversity Group, Department of Genetics Stellenbosch University Stellenbosch South Africa

**Keywords:** Ion Torrent, mitogenome, molecular divergence dating, phylogenetics, phylogeography

## Abstract

The Gempylidae (snake mackerels) family, belonging to the order Perciformes, consists of about 24 species described in 16 genera primarily distributed in tropical, subtropical, and temperate seas worldwide. Despite substantial research on this family utilizing morphological and molecular approaches, taxonomy categorization in this group has remained puzzling for decades prompting the need for further investigation into the underlying evolutionary history among the gempylids using molecular tools. In this study, we assembled eight complete novel mitochondrial genomes for five Gempylidae species (*Neoepinnula minetomai*, *Neoepinnula orientalis*, *Rexea antefurcata*, *Rexea prometheoides*, and *Thyrsites atun*) using Ion Torrent sequencing to supplement publicly available mitogenome data for gempylids. Using Bayesian inference and maximum‐likelihood tree search methods, we investigated the evolutionary relationships of 17 Gempylidae species using mitogenome data. In addition, we estimated divergence times for extant gempylids. We identified two major clades that formed approximately 48.05 (35.89–52.04) million years ago: Gempylidae 1 (*Thyrsites atun*, *Promethichthys prometheus*, *Nealotus tripes*, *Diplospinus multistriatus*, *Paradiplospinus antarcticus*, *Rexea antefurcata*, *Rexea nakamurai*, *Rexea prometheoides*, *Rexea solandri*, *Thyrsitoides marleyi*, *Gempylus serpens*, and *Nesiarchus nasutus*) and Gempylidae 2 (*Lepidocybium flavobrunneum*, *Ruvettus pretiosus*, *Neoepinnula minetomai*, *Neoepinnula orientalis*, and *Epinnula magistralis*). The present study demonstrated the superior performance of complete mitogenome data compared with individual genes in phylogenetic reconstruction. By including *T. atun* individuals from different regions, we demonstrated the potential for the application of mitogenomes in species phylogeography.

## Graphical abstract

The Gempylidae mitogenomes present a conserved gene order and a similar manner of evolution. Complete mitogenome data provide clearer resolution in the phylogeny of gempylids. We identify two major clades of the Gempylidae, which formed around 48.05 (35.89–52.04) million years ago.

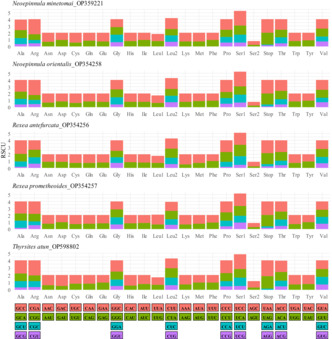

## 1 INTRODUCTION

The Gempylidae fishes commonly known as snake mackerels consist of 24 species described in 16 genera of which 13 are monotypic (Nakamura & Parin, 1993; Nelson, 2006). Gempylids are widely distributed in deeper tropical and subtropical waters (Blend et al., 2010; Li, Li, et al., 2020; Li, Qi, et al., 2020). A number of these species are economically important (Bustamante & Ovenden, 2016; Carnevale, 2006; Griffiths, 2002). Snake mackerels belong to the taxonomically complex suborder Scombroidei, and together with trichiurids (Trichiuridae) they form the Trichiuroidae superfamily (Nakamura & Parin, 1993). Gempylids and trichiurids are among the best‐known fishes anatomically and key distinguishing features for these families exist, that is, the number of nostrils, presence/absence of caudal fin, and morphology of dorsal fins (see Nakamura & Parin, 1993). Although they are treated as separate groups, most authors have presented evidence that the trichiurids represent a subgroup of gempylids (Carpenter et al., 1995; Johnson, 1986; Monsch, 2000). This may be due to the fact that several crucial family diagnostic characteristics are not uniformly distributed within each family (Carnevale, 2006). For example, the conical process on the tip of the upper/lower jaw only occurs in some species of the gempylids or trichiurids, or the anterior soft ray fins, which are underdeveloped or absent in many species of the Trichiuroidae (Nakamura & Parin, 1993). Numerous studies have discussed the systematics of these fishes and their relationships (Carpenter et al., 1995; Collette, 1984; Gago, 1997; Johnson, 1986; Monsch, 2000).

Challenges of taxonomic classification within the Trichiuroidae extend beyond superfamily level to family level. While gempylids can be identified by several characteristics considered as diagnostic for the family and the assignment of species to their genera does not seem to be in question, it is not always clear whether some genera belong to a certain tribe or another (Carnevale, 2006; Monsch, 2000; Nakamura & Parin, 1993). The variations between genera are minor and subtle, prompting questions about whether they are significant enough to warrant separation at the genus level or should be viewed as indicators of interspecific variation. Furthermore, it has been shown that some meristic traits used to designate species within the Gempylidae change with species' geographic range (Collette, 1984; Grey, 1953; Mikhailin, 1983; Parin et al., 1978; Parin & Bekker, 1972).

Although different traits for identifying specimens to species exist, the evolutionary histories of these attributes are unknown or poorly understood. As a result, past morphology‐based phylogenetic investigations have failed to establish the interrelationships of Gempylidae species (Carpenter et al., 1995). Including such morphological characters in the construction of the cladogram without consideration means the cladogram cannot provide independent reference for the interpretation of family evolution but for character evolution (Block & Finnerty, 1994; Carpenter et al., 1995). To avoid this circularity some researchers have used molecular markers to understand the interrelationships of the complex gempylids (Miya et al., 2013; Orrell et al., 2006). While mitochondrial genome (mitogenome) genes such as *COI*, *ND*2, *12S rRNA*, *16S rRNA*, and *CYTB*, are routinely used in phylogenetic and population genetics studies (Liu et al., 2016; Meng, 2019; Orrell et al., 2006; Schroeter et al., 2020; Yoon & Park, 2015), it is well known that individual genes independently often fail to properly resolve phylogenetic trees (Iwasaki et al., 2013). Whole mitogenomes, on the other hand, offer more understanding and finer resolution from higher‐level classification (i.e., order and family) down to closely related species than individual gene sections do (Miya & Nishida, 2000). Consequently, mitogenomic data have been widely employed to resolve complex phylogenetic relationships like for example in the Otocephala cohort (Lavoué et al., 2005) and in the Scombridae family (Friedman et al., 2019). Accordingly, a rapid increase in the number of fish mitogenomes published compelled the establishment of a whole database dedicated to fish mitogenomes. The MitoFish database (as of 08 May 2023) has 42,955 (species) complete and partial mitogenomes (http://mitofish.aori.u‐tokyo.ac.jp/), which is attributed to recent advances in high‐throughput sequencing methods, where greater throughput at reduced cost has enabled the quick discovery of mitogenomes.

Vertebrate mitochondrial genomes are double‐stranded circular molecules generally 16–19 kbp in size (Jondeung & Karinthanyakit, 2010; Mukundan et al., 2020; Prosdocimi et al., 2012). A typical vertebrate mitogenome encodes 13 protein‐coding genes (*ATPase 6* and *8*, *COI‐III*, *CYTB*, *ND1‐6*, and *4L*), two ribosomal RNA genes (*12S* and *16S*), 22 transfer RNAs (tRNAs), and two noncoding genes involved in replication and transcription, that is, the light‐strand replication origin (O_L_) and the highly variable displacement loop (D‐loop). The gene order in the mitogenomes is extremely conserved, with similar orders being observed/found in amphibians to mammals (Castro Antönia & Ramon, 1998; Satoh et al., 2016; Timbó et al., 2017). Nevertheless, duplication/deletion events (Bernt et al., 2013), loss of tRNAs (Liu & Cui, 2009; Mukundan et al., 2020), and variation in gene‐order arrangements have been reported in some vertebrate mitogenomes (Papetti et al., 2021; Satoh et al., 2016). These gene rearrangements, although not well understood (Satoh et al., 2016), along with other characteristics of mitogenomes, like the secondary structure of tRNA and mtDNA replication control models, are valuable for deep‐level phylogenetic studies in taxonomy (Liu et al., 2016; Mukundan et al., 2020; Papetti et al., 2021). Contrary to nuclear genomes, mitogenomes are typically homoplasmic, maternally inherited, and recombination‐free (Jondeung & Karinthanyakit, 2010; Mukundan et al., 2020). Additionally, they are more frequently substituted than nuclear genomes but are nevertheless substantially conserved, making them taxonomically discriminative.

In the current study, we characterized complete mitogenomes of *Neoepinnula minetomai* (Nakayama et al., 2014), *Neoepinnula orientalis* (Gilchrist & von Bonde, 1924), *Rexea antefurcata* (Parin, 1989), *Rexea prometheoides* (Bleeker, 1856), and *Thyrsites atun* (Euphrasén, 1791) species using the next‐generation sequencing (NGS) platform, Ion Torrent. Following that, we performed comparative analyses looking at gene content and gene‐order arrangement, codon usage, and nucleotide composition. Finally, we constructed phylogenetic trees using Bayesian inference and maximum‐likelihood methods and estimated time since the divergence of the Gempylidae family using 13 protein‐coding genes.

## 2 MATERIALS AND METHODS

### 2.1 Fish sampling and DNA extraction

**FIGURE 1:**
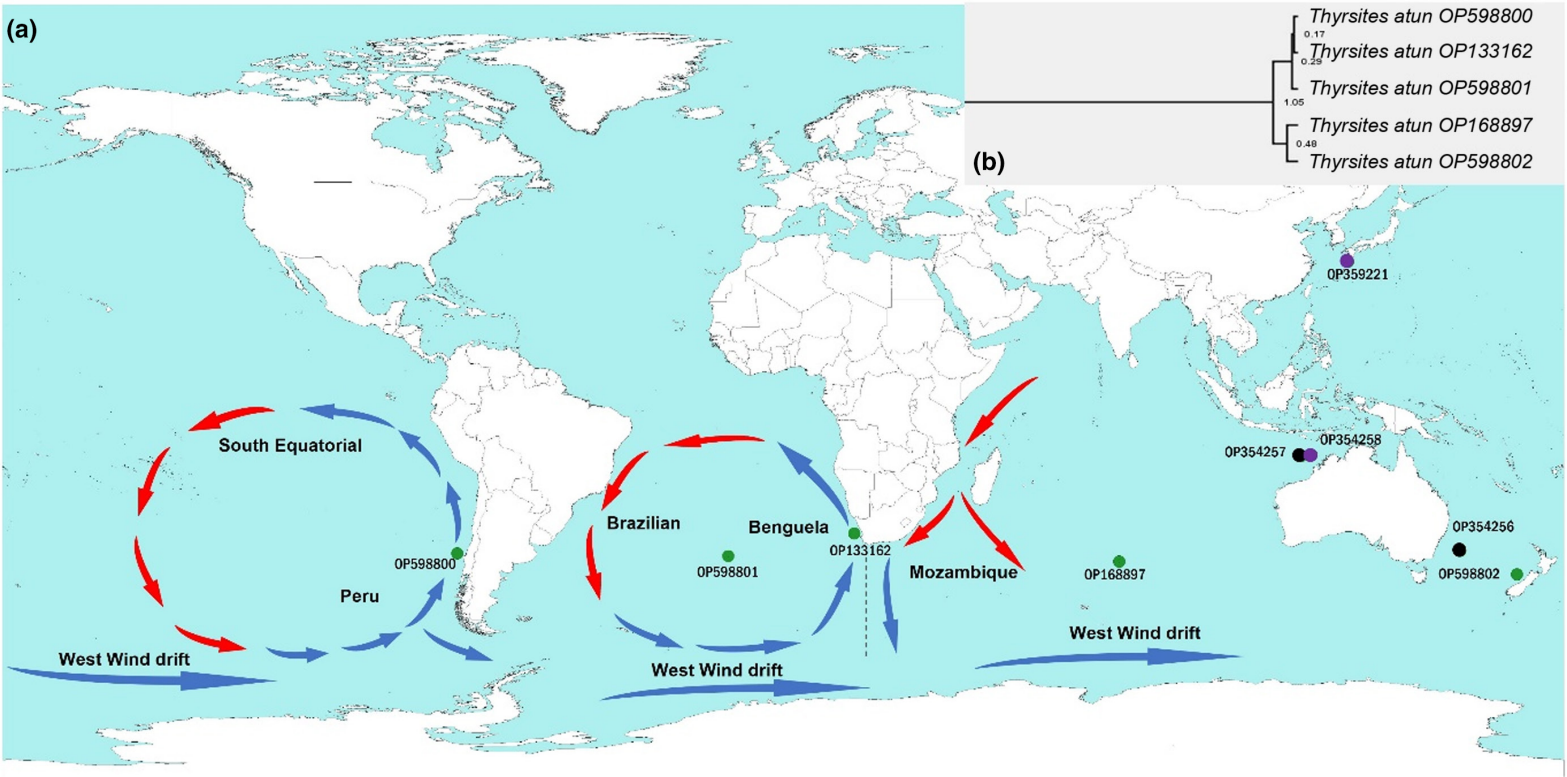
(a) Map showing sampling sites for five species of Gempylidae sequenced in the current study. The South African sample OP133162 included here was first reported by Mthethwa et al. (2023). Individuals of *Thyrsites atun* are represented by green disks, members of *Neoepinnula* by purple disks, and members of *Rexea* by black disks. The arrows represent directions of oceanic currents in the Southern Hemisphere (blue—cold and red—warm currents). The dotted vertical line indicates the barrier where the currents of the Atlantic and Indian oceans meet. (b) The clade of *Thyrsites atun* shows relationships among the members of *T. atun* studied here.

Tissue samples (fin clips and muscle) used in the current study were obtained from both local and international government fisheries and international research institutions. The fishes were originally caught during the 2014–2019 period from the following localities: *Neoepinnula minetomai* OP359221 (Kuchierabu‐Jima Island, Kagoshima, East China Sea: 30°28′12.5″N 130°07′49.4″E), *Neoepinnula orientalis* OP354258 (north‐west of Cape Leveque, Australia: 14°50′56.4″S 121°28′09.6″E), *Rexea antefurcata* OP354256 (Gascoyne Seamounts, Australia: 36°41′00.0″S 156°13′06.0″E), and *Rexea prometheoides* OP354257 (north‐west of Cape Leveque, Australia: 15°00′37.2″S 121°39′45.0″E). *Thyrsites atun* individuals were collected from four different geographic localities; OP598800 (Concepción, Chile: 36°51′11.5″S 73°19′33.8″W), OP598802 (west coast South Island, New Zealand: 41°35′46.0″S 170°46′46.0″E), OP598801 (Inaccessible Islands: 37°18′51.0″S 12°39′00.0″W), and OP168897 (Amsterdam and Saint‐Paul Islands: 38°41′33.0″S 77°31′33.6″E) (Figure 1). The tissue samples were preserved in 99% (v/v) ethanol until processing. Total genomic DNA (gDNA) was extracted from fin clips or muscle tissue using a standard cetyltrimethylammonium bromide (CTAB) extraction protocol (Sambrook & Russell, 2001).

### 2.2 Library construction and Ion Torrent sequencing

Sequencing was carried out at the Central Analytical Facility of Stellenbosch University, South Africa. Before library construction, gDNA concentration was quantified on the Qubit™ 4 Fluorometer, and the purity was determined using the NanoDrop™ ND‐2000 spectrophotometer (Thermo Fisher Scientific). Genome Quality Scores (GQS) were determined by electrophoresis using the PerkinElmer® LabChip GX Touch 24 Nucleic Acid Analyzer (PerkinElmer). Library preparation was performed using the Ion Plus Fragment Library Kit (ThermoFisher Scientific) according to the manufacturer's protocol, Ion Xpress™ Plus gDNA Fragment Library Preparation User Guide (Pub. No. MAN0009847 K.0). Following library preparation, template DNA was enriched using the Ion 530™ Chef Kit (ThermoFisher Scientific). Sequencing was carried out on the Ion GeneStudio S5 Prime System (Thermo Fischer Scientific). Post sequencing, raw reads were trimmed to remove the 3′ adaptor sequence in Torrent Suite v5.12.0 (Thermo Fischer Scientific). Further trimming using a 30 bp sliding window and a threshold value of 16 was performed. If the average quality value (QV) of the last 30 bases was lower than 16, the last 15 bases were trimmed away and the average quality score for the last 30 bases was calculated again and if needed, the last 15 bases were trimmed again. This process was repeated until the average QV for the last 30 bases was higher than 16. If the read was trimmed to less than 25 bases the whole read was removed from the dataset.

### 2.3 Mitochondrial genome assembly and annotation

Complete and partially complete mitogenomes of several Gempylidae species are available in NCBI's GenBank, therefore, a map‐to‐reference assembly method was used for most of the assemblies, with one exception where a combination of both the de novo and map‐to‐reference assembly methods was necessary. *Rexea antefurcata* and *R. prometheoides* sequence reads were mapped and assembled to *R. solandri* (KJ408216); *Neoepinnula minetomai* and *N. orientalis* raw reads were mapped to the closely related *Epinnula magistralis* (AP012943). As previously stated, four *T. atun* samples were sequenced, each from a distinct geographic location. The New Zealand sample with a mean length of 374 base pairs (bp) and 4,019,478 reads was considered the best candidate for draft genome assembly. *Thyrsites atun* NGS reads were mapped to a partially complete mitogenome of the closely related *Gempylus serpens* (AP012502) according to the study of Miya et al. (2013) using Geneious Prime v2020.2.5 (Biomatters Ltd.), resulting in an incomplete *T. atun* mitogenome missing the control region. To improve genome length coverage, a de novo assembly using the SPAdes v3.15.5 assembler (Prjibelski et al., 2020) was performed and the resulting contigs were aligned to the incomplete *T. atun* mitogenome. The contigs extended to the presumed control region, generating a full mitogenome. The newly constructed *T. atun* mitogenome was then utilized as a reference sequence for the remaining *T. atun* genome assemblies. Read mapping and assembly were carried out in Geneious Prime v2020.2.5 software (https://www.geneious.com) utilizing the Geneious mapper with medium/low sensitivity and fine‐tuning up to five iterations, followed by manual curation.

The position of the 13 protein‐coding genes (PCGs), 2 rRNAs, and 22 tRNAs was determined using MitoAnnotator (Iwasaki et al., 2013) and confirmed with the MITOS genome annotation pipeline (Bernt et al., 2013) using the vertebrate mitochondria genetic code. Annotations were performed in Geneious Prime and linear maps of complete mitogenomes were generated using the CGView server (https://proksee.ca). Protein‐coding sequences were translated into amino acids and checked for the presence of premature stop codons indicative of nuclear mitochondrial DNA segments (NUMTs). Nucleotide and amino acid composition, codon usage, and relative synonymous codon usage (RSCU) of the 13 protein‐coding genes were analyzed using MEGA v11 (Tamura et al., 2021). Nucleotide compositional skew was calculated according to the formula: AT‐skew = (A − T)/(A + T) and GC‐skew = (G − C)/(G + C) (Perna & Kocher, 1995). Information on the full characterization and nucleotide composition of these genomes can be found in Tables S1 and S2.

### 2.4 Gempylidae phylogeny

The phylogenetic relationship of 21 gempylids representing 17 species was investigated (Table 1). Thirteen PCG sequences excluding stop codons were aligned separately with ClustalX2 (Larkin et al., 2007) using default settings. The resulting alignments were optimized manually in BioEdit v7.2.5 (Hall, 1999) to remove regions that were challenging to align because of unique insertions into one or more sequence regions. The best‐fitting model for nucleotide substitution was determined in jModelTest v2.1.10 (Darriba et al., 2012) according to the Bayesian information criterion (BIC). Descriptive statistics of single gene alignments and their predicted models of evolution are presented in Table S3. Following model testing, the 13 alignments were concatenated in the order in which they appeared in the mitogenome. Bayesian inference (BI) and maximum‐likelihood (ML) were performed in MrBayes v3.2.7 (Ronquist et al., 2011) and Garli2.0 (Zwickl, 2008), respectively, using moderately gene‐partitioned data. A heuristic tree search was performed using ML and the confidence level of/for each branch was evaluated by performing bootstrapping with 100 replicates. The Markov Chain Monte Carlo (MCMC) analysis was run for 20,000,000 generations to allow for adequate time for convergence with sampling at every 1000 generations. Twenty‐five percent of starting trees were discarded as burn‐in while the remaining trees were used to estimate the consensus tree (50% majority rule) and the Bayesian Posterior Probabilities (BPP). To ensure that stationarity had been reached, the Effective Sample Size (ESS) for all sampling parameters was checked in Tracer v1.7 (Rambaut et al., 2018). The resulting trees obtained from the BI and ML analyses were visualized and edited in FigTree v1.4.4 (Rambaut, 2012).

**TABLE 1:** List of complete and partial mitochondrial sequences used in Gempylidae phylogenetic reconstruction. *Trichiurus lepturus* and *Benthodesmus tenuis* (Scombriformes: Trichiuridae) were used as outgroups.

Species	Family	Source	Accession number	Length (bp)	Status	Authors
*Diplospinus multistriatus* (Maul, 1948)	Gempylidae	NCBI	AP012523	16,650	Partial	Miya et al. (2013)
*Epinnula magistralis* (Poey, 1854)	Gempylidae	NCBI	AP012943	16,545	Complete	Miya et al. (2013)
*Gempylus serpens* (Cuvier, 1829)	Gempylidae	NCBI	AP012502	16,182	Partial	Miya et al., (2013)
*Lepidocybium flavobrunneum* (Smith, 1843)	Gempylidae	NCBI	AP012519	16,759	Partial	Miya et al. (2013)
*Nealotus tripes* (Johnson, 1865)	Gempylidae	NCBI	AP012521	16,197	Partial	Miya et al. (2013)
*Neoepinnula minetomai* (Nakayama et al., 2014)	Gempylidae	Current study	OP359221	16,529	Complete	
*Neoepinnula orientalis* (Gilchrist & von Bonde, 1924)	Gempylidae	Current study	OP354258	16,550	Complete	
*Nesiarchus nasutus* (Johnson, 1862)	Gempylidae	NCBI	AP012503	16,703	Complete	Miya et al. (2013)
*Paradiplospinus antarcticus* (Andriashev, 1960)	Gempylidae	NCBI	MN510443	16,988	Complete	Li, Li, et al. (2020), Li, Qi, et al. (2020)
*Promethichthys prometheus* (Cuvier, 1832)	Gempylidae	NCBI	AP012504	16,810	Complete	Miya et al. (2013)
*Rexea antefurcata* (Parin, 1989)	Gempylidae	Current study	OP354256	16,401	Complete	
*Rexea nakamurai* (Parin, 1989)	Gempylidae	NCBI	AP012520	16,382	Complete	Miya et al. (2013)
*Rexea prometheoides* (Bleeker, 1856)	Gempylidae	Current study	OP354257	16,386	Complete	
*Rexea solandri* (Cuvier, 1832)	Gempylidae	NCBI	KJ408216	16,350	Complete	Bustamante and Ovenden (2016)
*Ruvettus pretiosus* (Cocco, 1833)	Gempylidae	NCBI	AP012506	16,202	Complete	Miya et al. (2013)
*Thyrsites atun* (Euphrasen, 1791)	Gempylidae	NCBI	OP133162	16,494	Complete	Mthethwa et al. (2023)
*Thyrsites atun* (Euphrasen, 1791)	Gempylidae	Current study	OP598802	16,486	Complete	
*Thyrsites atun* (Euphrasen, 1791)	Gempylidae	Current study	OP598801	16,490	Complete	
*Thyrsites atun* (Euphrasen, 1791)	Gempylidae	Current study	OP168897	16,487	Complete	
*Thyrsites atun* (Euphrasen, 1791)	Gempylidae	Current study	OP598800	16,489	Complete	
*Thyrsitoides marleyi* (Fowler, 1929)	Gempylidae	NCBI	AP012505	16,138	Complete	Miya et al. (2013)
*Trichiurus lepturus* (Linnaeus, 1758)	Trichiuridae	NCBI	MK333401	16,840	Complete	Mukundan et al. (2020)
*Benthodesmus tenuis* (Günther, 1877)	Trichiuridae	NCBI	AP012522	16,864	Complete	Miya et al. (2013)

### 2.5 Molecular divergence dating

Molecular divergence dating was performed in BEAST v2.6.6 (Bouckaert et al., 2019). An alignment of 13 PCGs was codon‐partitioned to improve the data's phylogenetic signal. ModelFinder (Kalyaanamoorthy et al., 2017) implemented in IQ‐TREE v2.1.3 was used to select the best partitioning schemes for the data. A package in BEAST, bModelTest, was used to infer substitution models for the identified partitions (Bouckaert & Drummond, 2017). bModelTest package allows for model prediction during the MCMC analysis. Site models were inferred from a transversion/transition split subset (Table S4). The molecular phylogeny was calibrated using the fossil occurrence data of the most recent common ancestor (mrca) for *Thyrsitoides marleyi* and *Gempylus serpens*, *Thyrsitoides zarathoustrae*. This fossil was discovered in the fish‐producing strata of Istehbanât and Elam of the late Eocene to early Oligocene 28.1–33.9 million years ago (mya) (Friedman et al., 2019). Gempylidae‐specific speciation rates published by Rabosky et al. (2018) were used as birthRateY prior (Mean = 0.053, SD = 0.001). To retain species‐tree relationships while predicting branch lengths, a starting tree was used. This was achievable since the current study's Bayesian inference and maximum‐likelihood analysis yielded the same tree topology.

Prior to running the final analysis, we performed a series of tests to identify the appropriate parameters and priors for our data, such as testing the data for clock‐likeness and comparing the performance of tree priors, specifically the Calibrated‐Yule versus Birth‐Death Model, and the calibration priors (normal versus log‐normal distribution). The concluding MCMC analysis was conducted using the Calibrated‐Yule tree model together with the uncorrelated relaxed molecular clock model while applying a normal distribution calibration prior (Mean = 31, Sigma = 0.5). We performed two independent runs each comprising 200,000,000 iterations with sampling at every 5000 generations. Before merging the results of the two runs we inspected the output log files in Tracer v1.7.2 (Rambaut et al., 2018) to check: (i) for effective sampling of estimated parameters (ESS values > 200), (ii) to confirm both runs converged to the same posterior distribution (each run should be sampling from the same distribution). Once this was confirmed, 20% burn‐in was removed and the results were merged in LogCombiner v2.6.7 (Drummond & Bouckaert, 2015). A consensus tree (Maximum Clade Credibility Tree) summarizing the posterior tree distribution was constructed with TreeAnnotator v2.6.6 (Drummond & Bouckaert, 2015). The resulting tree was visualized and edited in FigTree v1.4.4 (Rambaut, 2012).

## 3 RESULTS

### 3.1 Genome composition

**FIGURE 2:**
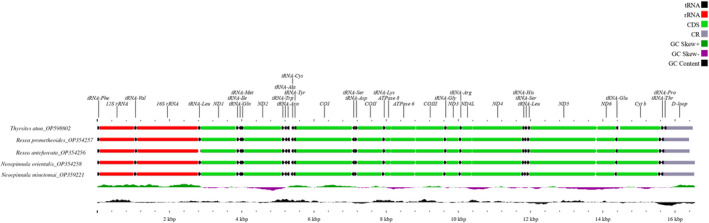
Complete linear mitochondrial genome maps of five Gempylidae species. Mitogenome features of *Neoepinnula orientalis* (OP354258), *Rexea antefurcata* (OP354256), *Rexea prometheoides* (OP354257), and *Thyrsites atun* (OP598802) are mapped to the largest contig of the five compared mitogenomes: that of *Neoepinnula minetomai* (OP359221). All mitogenomes expressed nearly identical nucleotide composition. Annotations were performed in Geneious Prime software and linear maps were generated in the CGView server.

The eight fish mitogenomes generated and compared in this study ranged between 16,401 and 16,550 bp in size, which falls within the expected range for most teleost mitogenomes. As in other vertebrates, all mitogenomes comprise 37 genes (13 protein‐coding, 22 tRNA, and 2 rRNA genes) plus the noncoding D‐loop region. The 37 genes were arranged in the same order as in typical vertebrate mitogenomes. Most genes were encoded on the heavy strand, except *ND6* and eight other tRNA genes (*tRNA* ^*Gln*^, *tRNA* ^*Ala*^, *tRNA* ^*Asn*^, *tRNA* ^*Cys*^, *tRNA* ^*Tyr*^, *tRNA* ^*Ser*^, *tRNA* ^*Glu*^, and *tRNA* ^*Pro*^), which occurred on the light strand. Two forms each of *tRNA* ^*Leu*^ and *tRNA* ^*Ser*^_,_ and the three tRNA clusters; IQM (Ile, Gln, and Met), WANCY (Trp, Ala, Asn, Cys, and Tyr), and HSL (His, Ser, and Leu) were identified, similar to other fish mitogenomes (Figure 2). The tRNAs varied from 65 to 76 bp in size, which is consistent with prior fish studies. Similarly, to other vertebrates, the 22 tRNA genes utilized the same anticodons across all five species.

### 3.2 Protein‐coding gene features

Three types of start codons were identified; ATG (*ATPase 6*, *ATPase 8*, *COII*, *COIII*, *ND1*, *ND2*, *ND3*, *ND4L*, *ND4*, *ND5*, and *CYTB*), GTG, and TAC, which were exclusive to *COI* and *ND6*, respectively. Seven types of stop codons were recognized, five of these were complete stop codons; TAA, AGG, AGA, ATT, and ATC, and the remaining two were incomplete stop codons (TA‐ and T‐‐). While most PCGs terminated with the TAA codon, *COII*, *ND3*, and *CYTB* terminated with an incomplete codon T‐‐, *ND2* terminated with an incomplete codon TA‐, and *ND4* used three stop codons: AGA, TA‐, and T‐‐.

### 3.3 Nucleotide composition

**FIGURE 3:**
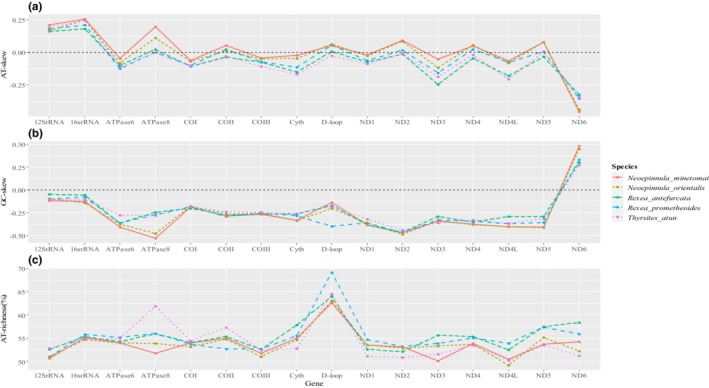
Nucleotide composition of *Neoepinnula minetomai* (OP359221), *Neoepinnula orientalis* (OP354258), *Rexea antefurcata* (OP354256), *Rexea prometheoides* (OP354257), and *Thyrsites atun* (OP598802) calculated per gene region using MEGA v.11 [(a) AT‐skew = (A−T)/(A + T); (b) GC‐skew = (G−C)/(G + C); (c) AT‐richness (%) = A (%) + T (%)].

The mitogenomes of the five species studied were generally AT‐rich, whether it was the protein‐coding, tRNA, or rRNA genes being investigated. The control region D‐loop had the highest A + T percentage (Table 2). Although protein‐coding genes were AT‐rich, they presented negative AT‐skew values in all five species: nucleotide T compared with A was overly represented. All mitogenomes, whether protein‐coding subsets, tRNA, rRNA genes, or control regions were considered, presented moderate negative GC‐skew values, which was expected (nucleotide C was overly represented over G) (Table 2). The AT‐richness (%) and nucleotide compositional skew per gene are presented in Figure 3. All 13 PCGs except for the *ND6* gene showed a negative GC‐skew. Table 3 shows sequence nucleotide distribution per codon position. Pronounced anti‐G bias was observed at the second codon positions. The A + T composition of the second codon position was relatively higher compared with 1st and 3rd codon positions, which is not uncommon with vertebrates. Detailed nucleotide composition is presented in Table S2.

**TABLE 2:** Nucleotide composition and skewness levels calculated for the sequenced majority strands of five Gempylidae species.

Species	A + T (%)				AT‐skew				GC‐skew			
	Mito	PCGs	rRNAs	D‐loop	Mito	PCGs	rRNAs	D‐loop	Mito	PCGs	rRNAs	D‐loop
*Neoepinnula minetomai* (OP359221)	54.2	53.6	53.6	62.6	0.08	−0.01	0.24	0.05	−0.29	−0.31	−0.12	−0.14
*Neoepinnula orientalis* (OP354258)	54.1	53.6	53.3	63.1	0.06	−0.02	0.22	0.06	−0.29	−0.31	−0.13	−0.20
*Rexea antefurcata* (OP354256)	55.3	55.0	54.3	64.0	0.01	−0.01	0.17	0.00	−0.25	−0.28	−0.06	−0.17
*Rexea prometheoides* (OP354257)	55.2	54.8	54.0	69.1	0.03	−0.06	0.20	0.05	−0.26	−0.29	−0.09	−0.40
*Thyrsites atun* (OP598802)	54.3	53.2	54.2	66.0	0.01	−0.08	0.23	−0.02	−0.25	−0.27	−0.11	−0.21

**TABLE 3:** Nucleotide composition of five Gempylidae mitogenomes partitioned into protein‐coding genes, transfer RNAs, and ribosomal RNAs.

	*Neoepinnula minetomai* (OP359221)				*Neoepinnula orientalis* (OP354258)				*Rexea antefurcata* (OP354256)				*Rexea prometheoides* (OP354257)				*Thyrsites atun* (OP598802)			
	T(U)	C	A	G	T(U)	C	A	G	T(U)	C	A	G	T(U)	C	A	G	T(U)	C	A	G
Proteins	27.0	30.4	26.6	15.9	27.3	30.3	26.2	16.1	30.0	28.9	25.0	16.1	29.0	29.2	25.8	16.0	28.9	29.7	24.3	17.1
1st	27.9	27.7	24.4	20.0	28.1	27.9	24.6	19.4	28.6	27.3	23.2	20.9	29.3	26.5	24.0	20.2	26.0	26.7	25.1	22.2
2nd	32.0	32.0	24.9	11.0	32.2	31.8	24.6	11.4	35.0	28.5	23.7	12.9	33.0	31.5	24.0	11.5	37.3	29.7	20.4	12.6
3rd	21.2	31.6	30.4	16.8	21.8	31.3	29.5	17.4	26.5	30.9	28.3	14.4	24.7	29.6	29.3	16.4	23.2	32.8	27.5	16.4
tRNAs	26.9	21.4	28.0	23.7	27.3	30.3	26.2	16.1	26.9	21.5	28.2	23.4	27.1	21.5	27.4	24.0	28.4	20.4	28.2	23.0
rRNAs	20.4	26.1	33.2	20.3	20.8	26.3	32.5	20.4	22.5	24.1	31.8	21.6	21.7	25.0	32.4	21.0	20.9	25.5	33.2	20.5

### 3.4 Codon usage and RSCU

**FIGURE 4:**
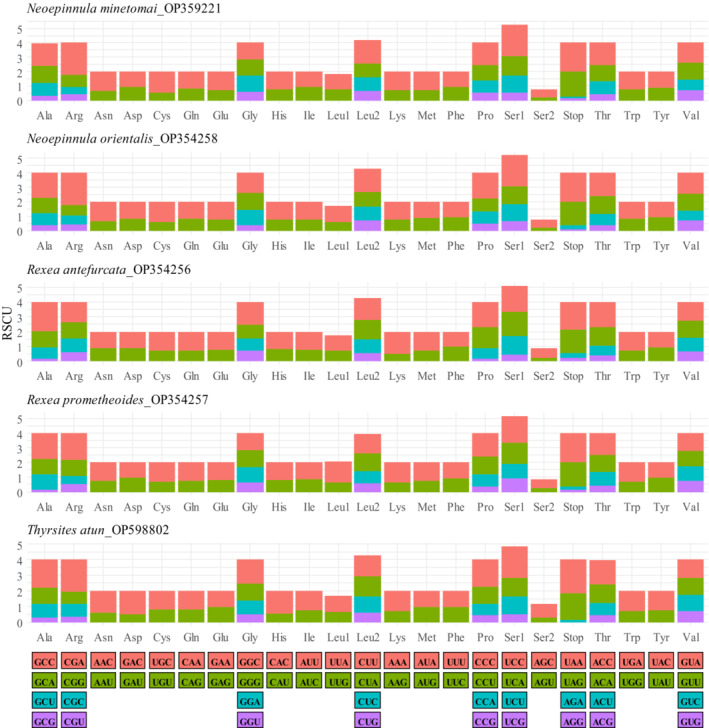
Relative Synonymous Codon Usage (RSCU) profile of *Neoepinnula minetomai* (OP359221), *Neoepinnula orientalis* (OP354258), *Rexea antefurcata* (OP354256), *Rexea prometheoides* (OP354257), and *Thyrsites atun* (OP598802) calculated in MEGA v11 using concatenated protein‐coding genes including stop codons.

Codon usage analyses revealed that Gempylidae fishes used all 64 codons to encode all 20 amino acids in their PCGs. Leucine, averaging 650 counts was the most frequent amino acid, whereas Cystine (averaging 42 counts) was the least common (Figure S1). Relative synonymous codon usage (RSCU) is an important measure of codon usage bias. The RSCU is defined as the ratio of the observed to the predicted frequency of codons when all synonymous codons for the same amino acid are utilized equally. If the RSCU value is higher than 1.0, there is a positive codon‐usage bias, and if the value is less than 1.0, there is a negative codon‐usage bias. Values higher than 1.6 and lower than 0.6 indicate overrepresented and underrepresented codons, respectively. Relative synonymous codon usage analysis for the five species indicated the most frequently used codons were codons ending in A/C (A‐ending > C‐ending), while codons ending in G/U were penalized (Figure 4).

### 3.5 Mitogenome length variation

Mitogenome sizes varied among individuals in the current study, both at genus (*Rexea* spp. an 87 bp difference, *Neoepinnula* spp. a 21 bp difference) and species level (*Thyrsites atun*, a minimum of 4 bp difference). Size variation was largely due to, (i) the different lengths of the noncoding D‐loop region (ranging from 603 nucleotides to 862 nucleotides), and (ii) the presence of intronic regions (these ranged from 1 to 54 bp). The mitogenome of *N. orientalis* was the largest of the eight samples studied and had the most introns. Gene length (PCGs and tRNAs) was similar across the examined five species except for *12S* rRNA, *16S* rRNA, and COI, which showed some slight variation. The smallest genome of *R. antefurcata* (16,401 bp) featured two shorter tRNAs; *tRNA* ^*‐Ser*^ and *tRNA* ^*‐Trp*^ of 53 and 62 bp, respectively, compared with the 71 bp seen in other gempylids or reported for other fishes. Some overlap was observed among protein‐coding genes located directly adjacent to each other whether the gene was encoded on the same strand or opposite strands.

### 3.6 Gempylidae phylogenetics

**FIGURE 5:**
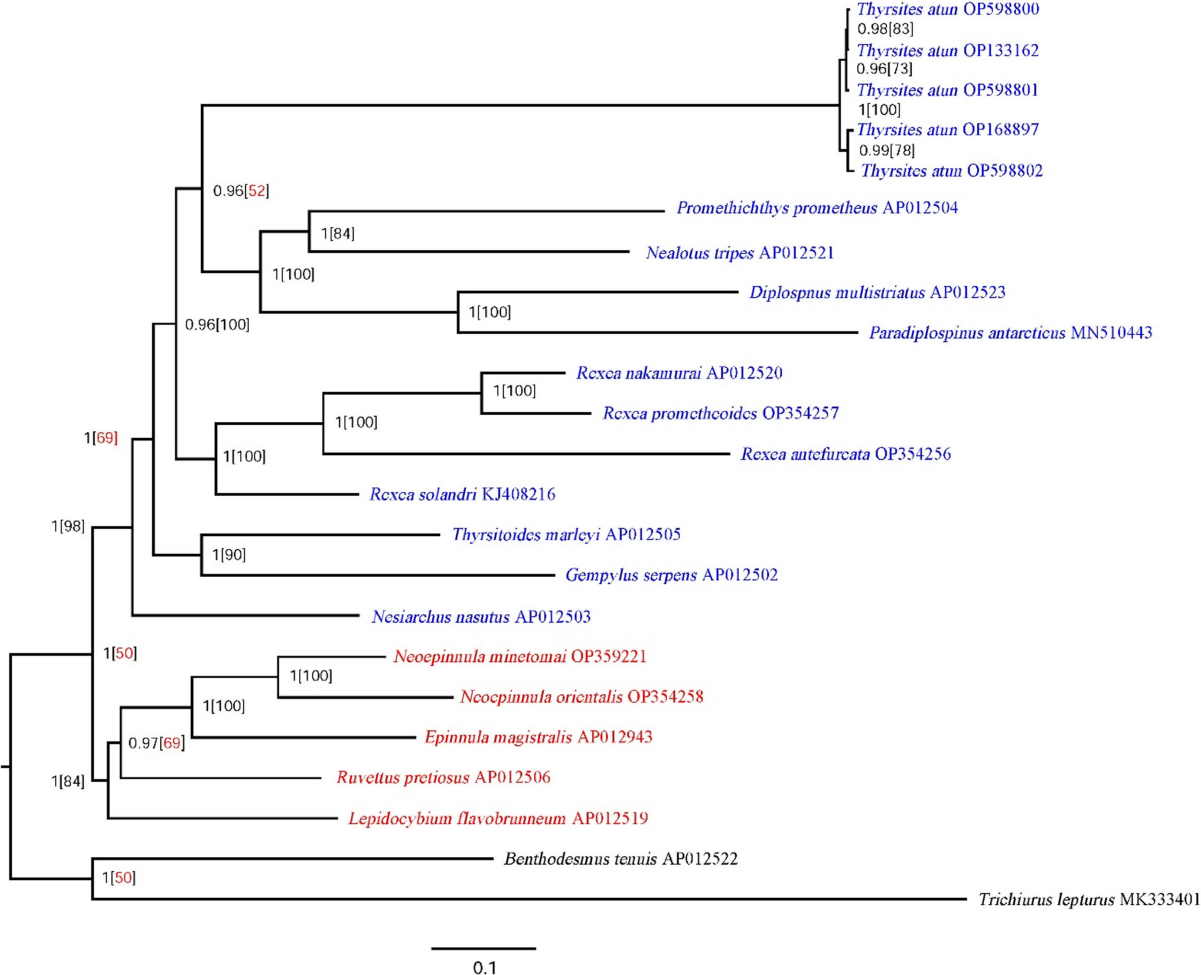
Gempylidae phylogeny reconstructed using the Bayesian inference and maximum‐likelihood methods from concatenated dataset of 13 protein‐coding genes of the mitochondria. Members of the trichiurids, *Benthodesmus tenuis* AP012522 and *Trichiurus lepturus* MK333401 were included as outgroups. The numbers on branches represent Bayesian Posterior Probabilities (BPP)/Bootstrap values (Bootstrap values below 70 are shown in red).

Both BI and ML tree search methodologies yielded nearly identical and well‐resolved phylogenetic trees using a concatenated dataset of 13 PCGs. There were a few differences in the node support metrics used by the two approaches, with bootstrap generally providing less support than Bayesian posterior probability. Bayesian Inference identified two primary clades within the Gempylidae family (Figure 5).

### 3.7 Estimating time since divergence

**FIGURE 6:**
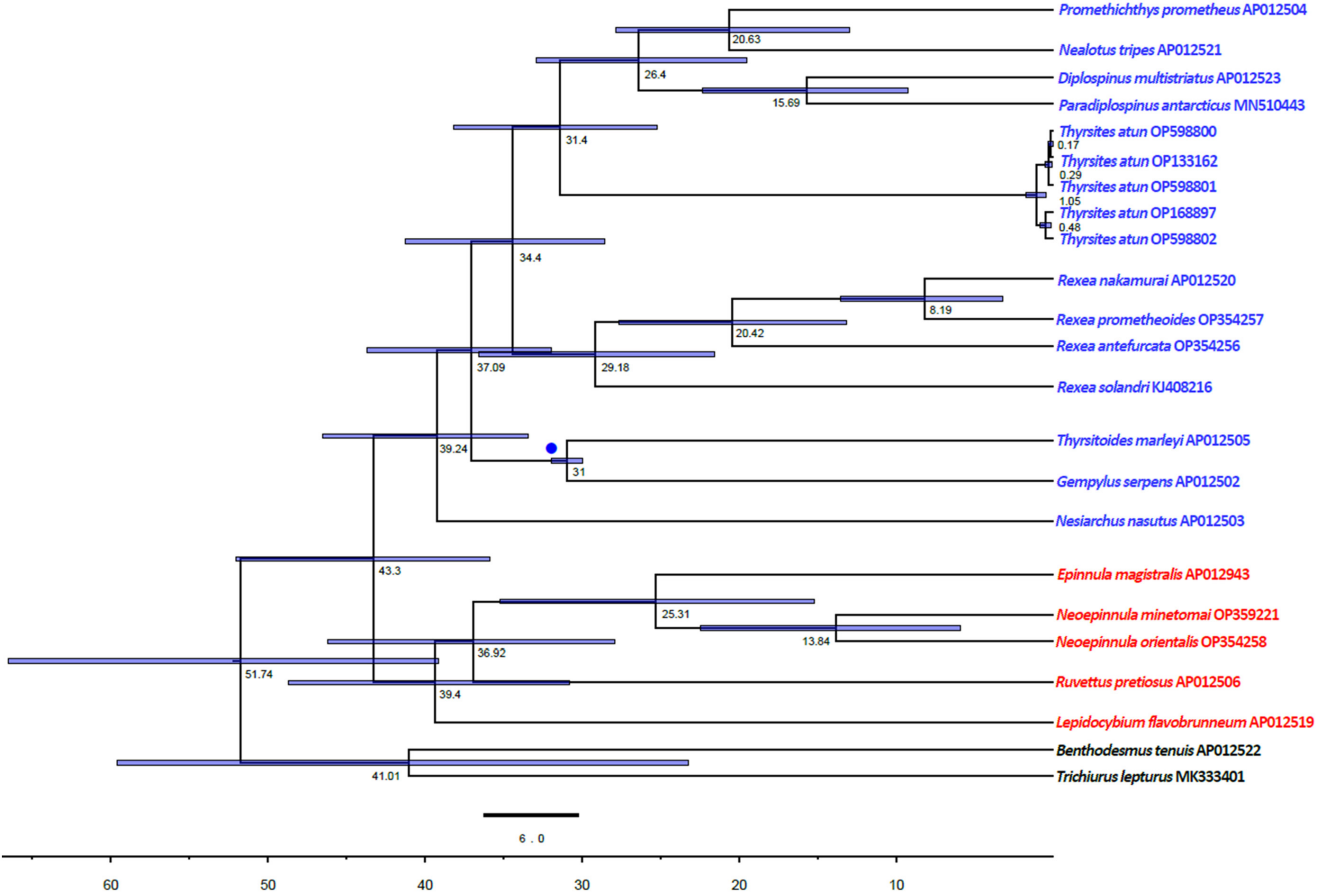
Time‐calibrated phylogenetic tree of 17 Gempylidae species. Mean divergence times were estimated using a relaxed molecular clock model on a subset of mitochondrial genes. Two species of trichiurids, *Benthodesmus tenuis* AP012522 and *Trichiurus lepturus* MK333401 were included as outgroups. The blue disk denotes the placement of the fossil calibration.

The two primary clades of the Gempylidae are estimated to have diverged around 43.3 mya (95% HPD: 35.89–52.0), while Gempylidae 1 and Gempylidae 2 diverged around 39.2 mya (95% HPD: 33.45–46.54) and 39.4 mya (95% HPD: 30.78–48.7), respectively (Figure 6). The minor clade of *P. prometheus*, *N. tripes*, *D. multistriatus*, and *P. antarcticus* nested within Gempylidae 1 is at least 28.5 million years old (95% HPD: 19.5–32.9). Notable divergence can be seen within individuals of *T. atun*, where two lineages exist for this group diverging at 1.05 mya (95% HPD: 0.5–1.77).

## 4 DISCUSSION

Advances in high‐throughput sequencing technologies have led to increased availability of genome‐scale data for evolutionary analysis. These data provide a great opportunity for studying genetic diversity, population dynamics, and evolutionary genetics. Here we characterized the complete mitochondrial genomes for five Gempylidae species using Ion Torrent sequencing. The mitogenomes varied in size from 16,401 to 16,550 bp, which was well within the predicted range for teleost mitogenomes (Liu & Cui, 2009). Gene content and order of arrangement were conserved as typically found in other vertebrates. Most genes were encoded on the heavy strand, except *ND6* and eight other tRNA genes, which occurred on the light strand. All eight mitogenomes were AT‐rich, a feature common to all teleost mitogenomes.

### 4.1 Protein‐coding gene features

A typical ATG initiation codon was found in all protein‐coding genes except *COI*, which exclusively used GTG, similar to most teleosts (Li et al., 2019), as a result, *COI* is thought to evolve at a much slower rate than other mitochondrial genes (Li et al., 2019). While TAA was the preferred termination codon for most protein‐coding genes, a pattern was observed across the five compared species, where *COII*, *ND3*, and *CYTB* terminated with an incomplete T‐‐ codon, *ND2* terminated with TA‐, *ND6*, which is coded on the opposite strand used ATT/ATC, while *ND4* used three stop codons; AGA, TA‐, and T‐‐, suggesting *ND4* and *ND6* might undergo a rapid evolutionary process in the Gempylidae species. The incomplete stop codons were mainly used when the 3′‐end of the protein‐coding genes (*ND2*, *COII*, *ATPase 6*, *COIII*, *ND3*, *ND4*, *CYTB*) was followed by a tRNA gene transcribed on the same strand. The presence of a tRNA gene, which serves as a punctuation marker, may allow transcription to end without using complete stop codons. During RNA processing, these stop codons can be converted to TAA by adding a poly‐A tail (Satoh et al., 2016). On the contrary, only complete stop codons were used in the *ND5*, *ND6*, and *COI* genes. These genes were followed by a gene encoded on the opposite strand, which causes transcription without punctuation and may explain the exclusive use of complete stop codons by these genes (Li, Li, et al., 2020; Li, Qi, et al., 2020; Satoh et al., 2016).

### 4.2 Mitogenome evolution

In fish, the mitochondria organelle plays an important role in ion regulation and the production of adenosine triphosphate (ATP) to maintain cell functioning and heat regulation. In essence, the mitochondrion allows fish to adapt, to some extent, to changes in water temperatures and salinity (Gerber et al., 2020; Lin & Sung, 2003). The fishes of the Gempylidae family have a widespread range, occurring in all ocean basins with varying gradients of salinity and water temperature, suggesting that various selective pressures are working on their mitogenomes. Therefore, their mitogenomes have the potential to be employed as a proxy for modeling evolutionary connections within the gempylids. Analysis of species codon usage profiles here quantified by codon count (Figure S1) and relative synonymous codon usage (Figure 4) revealed mitogenomes of these fishes followed similar evolution patterns.

Gempylids are among the best‐known fishes anatomically. However, until recently, taxonomic classification within the Gempylidae family remained controversial and a subject of continued interest in biological systematics. A morphological study of the gempylids by Russo (1983) identified two unresolved groups (Group1: *Nealotus*, *Rexea*, and *Promethichthys*, and Group2: *Thyrsites*, *Thyrsitops*, *Tongaichthys*, *Neoepinnula*, and *Epinnula*). A study by Gago (1997) using larval development recovered two unresolved groups (Group1: *Diplospinus*, *Epinnula*, *Paradiplospinus*, *Neoepinnula*, and *Rexea*; Group2: *Thyrsite*s, *Thyrsitops*, *Gempylus*, *Nesiarchus*, *Promethichthys*, and *Ruvettus*). Numerous similar studies have been conducted, all of which have produced different results (Carpenter et al., 1995; Collette, 1984; Johnson, 1986; Matsubara & Iwai, 1952). Many of these studies, if not all of them, made use of features that were either common within the family or diagnostic of one or more genera. Such features cannot be utilized to infer relationships between the genera. In the current study, we demonstrated the superior performance of concatenated mitochondrial protein‐coding genes in resolving complicated phylogenetic relationships such as for the Gempylidae. By utilizing multiple molecular markers our understanding of the evolutionary connections between species within the family was improved.

In the present study, gempylids were divided into two likely significant clades: Gempylidae 1 (*Thyrsites*, *Promethichthys*, *Nealotus*, *Diplospinus*, *Paradiplospinus*, *Rexea*, *Thyrsitoides*, *Gempylus*, and *Nesiarchus*), and Gempylidae 2 (*Lepidocybium*, *Ruvettus*, *Neoepinnula*, and *Epinnula*) (Figure 5). The results of the Bayesian inference and maximum‐likelihood approaches were considerably more in agreement, despite differences in node support. This is because although posterior probabilities and bootstraps are both approaches used to evaluate node support, these two metrics model different parameters, and as a result, disparities are not uncommon (Svennblad et al., 2006). Bayesian Inference is more conservative and less prone to strongly supporting a false phylogenetic hypothesis (Brooks et al., 2007; Douady et al., 2003).

According to Russo (1983) and Collette (1984), *Promethichthys*, together with *Nealotus*, and *Rexea*, are regarded as a monophyletic group within the gempylids and the genus *Diplospinus* is thought to be closely related to the genera *Gempylus*, *Nesiarchus*, and *Paradiplospinus* (Carpenter et al., 1995; Gago, 1997; Parin & Bekker, 1972). In the current study, these groups were nested within the Gempylidae 1 clade, which also contains *Thyrsites*, contrary to the classification of Russo (1983) where *Thyrsites* was grouped with *Neoepinnula* and *Epinnula* (Gempylidae 2 in the current study). Interestingly, it was in the study of Russo (1983) that the shared bone morphology between *Thyrsites*, *Diplospinus*, *Paradiplospinus*, *Promethichthys*, and *Nesiarchus* was identified. A study by Miya et al. (2013) based on *ND2* reported *T. atun* as sister taxa of *G. serpens*. This finding is in line with the results of our recent work, in which we used all 13 PCGs of mitochondria to investigate the placement of *T. atun* within the Gempylidae family Mthethwa et al. (2023). However, the current results indicate that *T. atun* and *T. marleyi* are not closely related, even though we remain convinced in the inclusion of *Thyrsites* in Gempylidae 1. This could be a result of unrepresentative sampling in the latter study that only investigated the relationship between 13 gempylids while the former study likely suffered from decreased phylogenetic resolution capability of individual genes. *Thyrsites atun*, also known as snoek and *Thyrsitoides marleyi* commonly known as black snoek share several morphological similarities. Furthermore, *Thyrsites* and *Gempylus* (true sister genus of *Thyrsitoides*) are the only members of the Gempylidae to share a caringiform swimming mode and a high number of dorsal and anal finlets, making them evolutionary close relatives (Monsch, 2000). *Lepidocybium flavobrunneum*, which has consistently been identified as the most primitive member of the gempylids, was found in the basal cluster as were *Ruvettus* and *Epinnula*. The placement of *Neoepinnula* within Gempylidae clade 2 was expected*. Neoepinnula* spp. and *Epinnula* spp. share numerous similarities and were previously assumed to be members of the same genus. *Nesiarchus nasutus* is the oldest member of Gempylidae 1. Our findings are congruent with the molecular classification of Jondeung and Karinthanyakit (2010), and the molecular phylogenetic studies of Friedman et al. (2019), Li, Li, et al. (2020), and Li, Qi, et al. (2020).

### 4.3 Evolution of Gempylidae—Calibrated time tree

The appearance of gempylids, scombrids, trichiuroids, and other distantly related predatory percomorphs in the early Paleogene is interpreted as a response to the extinction of major Mesozoic groups of predatory marine teleosts (Miya et al., 2013). The oldest Gempylidae fossil occurred in the Ypresian age (47.8–56.0 mya), an early era of the Eocene; the period after the Cretaceous–Paleogene (K–Pg) mass extinction (Alfaro et al., 2018; Beckett et al., 2018). Our result estimates the most recent common ancestor of the gempylids to be at least 43.3 (95% HPD: 35.89–52.04) million years old (Figure 6). The maximum age estimates for most clades confirm the findings of Friedman et al. (2019) but our minimum clade age estimates differ. Because we included more species in our analysis, our 95% HPD ranges were more conservative and potentially more reliable. Although the first reported fossil of Gempylidae comes from the Ypressian, our result showed modern‐day gempylids originated from the period after the early Eocene and mid‐Miocene with most diversification taking place between 20 and 40 mya (Figure 6) about 20 million years after the K‐Pg mass extinction. While the late Cretaceous and early Cenozoic mass extinction of K‐Pg is regarded as the fundamental cause of contemporary fish diversification (Alfaro et al., 2018; Li, Li, et al., 2020; Li, Qi, et al., 2020), it is reasonable to believe that a different geological event was responsible for the observed diversity in the Gempylidae family. The tectonic plate movement during the mid/late Eocene–Oligocene is one occurrence that might be related to the observed Gempylidae radiation. Tectonic plates are potentially one of the main determinants of marine biodiversity, as they modulate the distribution of seafloors through continental movement and collision over geological timescales, in turn shaping the marine food webs (Leprieur et al., 2016; Renema et al., 2008). Events linked to the shift of tectonic plates such as changes in ocean temperature, and oceanic currents have also been reported to influence biodiversity dynamics (Leprieur et al., 2016).

The gempylids and closely related sister groups such as scombrids are believed to have evolved from a deep‐ocean ancestor (Miya et al., 2013). Primitive gempylids such as *L. flavobrunneum*, *N. nasutus*, *R. pretiosus*, *G. serpens*, *T. atun*, and *T. marleyi*, occur abundantly in a wide range of depths (www.fishbase.org, version 02/2023). All these gempylids are distributed globally except for *T. atun* (restricted to the southern hemisphere) and *T. marleyi* found only in the Indian and Pacific oceans. By contrast, modern gempylids, such as *P. antarcticus*, *Rexea* spp., *Neoepinnula* spp., and *Epinnula* spp. show localized distribution mainly in shallower waters off the coasts of the Indian and Pacific oceans except for *P. prometheus*, *N. tripes*, and *D. multistriatus*, which are distributed globally. An interesting pattern of radiation can be seen within the *Rexea* genus. *Rexea solandri*, the primitive member of this genus, occurs only on the coasts of Australia and New Zealand. *Rexea antefurcata*, the second oldest *Rexea* species, is found along the east coast of Australia and the Tasman Sea, as well as in the west Pacific Ocean. The youngest pair of *Rexea* included in this study, the two sister taxa *R. prometheoides* and *R. nakamurai* occur in the Indian and the Pacific oceans. The recently diverged species have increased dispersal ability as opposed to the primitive *R. solandri* although still within the Indian and Pacific oceans.

### 4.4 Snoek phylogeography

While mitogenomes are routinely used for phylogenetic assessments, they are also invaluable resources for phylogeographic studies. In the current phylogenetic analyses, we included *T. atun* from five different geographic locations (Figure 1), and the results showed the existence of two lineages: (i) Amsterdam and Saint‐Paul Islands—New Zealand, and (ii) Inaccessible Islands—Chile—South Africa. Some geographic differentiation of *T. atun* was previously reported by Cawthorn et al. (2012) and later confirmed by Hüne et al. (2021). According to the latter study, *T. atun* first appeared on the coast of Chile and South Africa before moving on to those of Australia and New Zealand. On the contrary, our results show the ancestor of the *T. atun* lineage, at least 0.48 million years old, may have originated from the South‐Pacific (New Zealand) and Southern Indian (Amsterdam and Saint‐Paul Islands) oceans and later spread to the East‐Pacific and Atlantic oceans (Figure 6). A comparison of South African *T. atun* and that from the Amsterdam and Saint‐Paul Islands revealed that these two individuals were distantly related (genetic distance of 0.01910, 4998 kilometers apart). Interestingly, in the comparison of *T. atun* from the Amsterdam and Saint‐Paul Islands and from the Tasman Sea (New Zealand) a genetic distance of *K‐2‐P* = 0.01075 (7833 km apart) was obtained (Figure 1 and Table S5). These findings imply that currents, rather than the physical distance between the two lineages, significantly influence the gene flow of this species. The South African *T. atun* population occurs along the South Atlantic Ocean separated from the Indian Ocean by a barrier formed where the Benguela and Agulhas currents meet (Figure 1). This barrier plays a prominent role in limiting the dispersal of southern African coastal fishes (Maduna et al., 2016). Where two ocean currents converge, strong environmental gradients, such as temperature and salinity, have been observed. These gradients are believed to affect some mobile species' dispersion patterns and contribute to the genetic structuring of marine communities (Miller et al., 2013). This explains why *T. atun* from South Africa (Atlantic Ocean) versus *T. atun* from Amsterdam and Saint‐Paul Islands, although in geographic proximity to each other, but occurring on either side of the barrier, showed greater genetic variation than *T. atun* from Amsterdam and Saint‐Paul Islands versus *T. atun* from New Zealand, which are geographically farther apart but are found on the same side of the barrier (Indian and Pacific oceans).

## 5 CONCLUSION

Although short‐read mapping against a single reference sequence is a frequently used method for reconstructing genomes like in the present study, there is reason to believe that this method could introduce biases depending on the reference used for mapping (Valiente‐Mullor et al., 2021). The majority of these errors result from genetic differences between the reference and read sequence data, and they can have an impact on the analysis that follows (Valiente‐Mullor et al., 2021). The Ion Torrent, like all second‐generation sequencing technologies, is incapable of sequencing long stretches of DNA; these short‐read sequences frequently fail to generate sufficient overlap sequence from the DNA fragments. This constitutes a significant challenge for the de novo genome assembly (Adewale, 2020). The authors advocate the use of third‐generation sequencing technologies, long‐read sequencing technologies (LSR) as they generate a reasonable length of overlap sequence for better sequence assembly (Adewale, 2020).

The current study sought to investigate the utility of mitochondrial genome data in understanding the phylogenetic relationships of the taxonomically complex Gempylidae family. Although comparative analysis of Gempylidae mitogenomes revealed a high degree of similarity at the nucleotide and amino acid level, including gene arrangement and gene content, their potential to resolve the gempylids' phylogenetic relationships with higher confidence support than any other marker used previously, is remarkable. Even though this is the largest study to date to investigate the phylogenetic relationships of 17 (of 24 species), we were still unable to draw firm conclusions about the evolutionary history of the Gempylidae family because not all species/genera were represented (or included). Although there are several traits that are thought to be diagnostic for/in the family, some of them have either been misinterpreted or are not useful in determining the evolutionary position of the Gempylidae members. Our findings demonstrate the potential of employing information from all 13 PCGs of the mitochondrial genome to guide the careful selection of phylogenetically informative features. Our findings warrant further investigation of *T. atun* phylogeography.

## AUTHOR CONTRIBUTIONS

**Siphesihle Mthethwa:** Data curation (lead); formal analysis (lead); investigation (lead); methodology (lead); writing – original draft (lead). **Aletta E. Bester‐van der Merwe:** Conceptualization (equal); supervision (equal); writing – review and editing (equal). **Rouvay Roodt‐Wilding:** Conceptualization (equal); funding acquisition (equal); resources (equal); supervision (equal); writing – review and editing (equal).

## FUNDING INFORMATION

This work was supported by the National Research Foundation, South Africa under Grant (CPRR160425162975). The funding source was not involved in the study design, in the collection, analysis, and interpretation of data, in the writing of the report, and in the decision to submit the article for publication.

## CONFLICT OF INTEREST STATEMENT

The authors report there are no competing interests to declare. Submission declaration and verification: The work described in this article has not been published previously nor is it under consideration for publication elsewhere. Publication of this work is approved by all authors and explicitly by the responsible authorities where the work was carried out, and that, if accepted, it will not be published elsewhere in the same form, in English or any language, including electronically without the written consent of the copyright holder.

## Supporting information


Appendix S1

Table S1

Table S2

## Data Availability

The genome sequence data that support the findings of this study are openly available in GenBank of NCBI at https://www.ncbi.nlm.nih.gov/ under the following accession numbers: OP168897, OP354256–OP354258, OP359221, and OP598800–OP598802. The associated SRA PRJNA895979 and Bio‐Sample numbers are SAMN31528370‐77. Supporting material, including mitogenome characterization and input files for phylogenetic and selection analyses, is available in the Mendeley Data repository under DOI: 10.17632/z5vvwvjhzx.1.
