# Supplementary material for: Addressing the complex phylogenetic relationship of the Gempylidae fishes using mitogenome data
Source: Ecol Evol. 2023 Jun 21;13(6):e10217. doi: 10.1002/ece3.10217 (PMC10283032; doi:10.1002/ece3.10217)
Supplement: Supplementary file 1 — Appendix S1 [file ECE3-13-e10217-s003.docx]

**Addressing the complex phylogenetic relationship of the Gempylidae fishes using mitogenome data.**

Author(s): Siphesihle Mthethwa ^*^, Aletta van der Merwe, and Rouvay Roodt-Wilding.

*Molecular Breeding and Biodiversity Group, Department of Genetics, Stellenbosch University, Stellenbosch, South Africa*

**Corresponding author, e-mail:* [*sihlemthethwa@sun.ac.za*](mailto:sihlemthethwa@sun.ac.za)

JC Smuts Building, De Beer Rd, Stellenbosch Central, Stellenbosch, 7600.

Table of Contents

[**Table S1.** Main features of the complete mitochondrial genomes of five Gempylidae species; *Neoepinnula orientalis*, *Neoepinnula minetomai*, *Rexea antefurcata*, *Rexea prometheoides,* and *Thyrsites atun*. H-strand–heavy strand, L-strand–light strand; IGN–intergenic regions (+) and overlapping nucleotides (-). n/a. - not applicable. Intergenic spaces were counted manually. 1](#_Toc136558137)

[**Table S2.** Nucleotide composition calculated for the major strand for five Gempylidae species. 2](#_Toc136558138)

[**Table S3.** Gene alignment characteristics estimated on MEGA v11, and their corresponding substitution models as predicted by jModelTest. 2](#_Toc136558139)

[**Figure S1.** Codon usage profile of *Neoepinnula minetomai* [OP359221]*, Neoepinnula orientalis* [OP354258]*, Rexea antefurcata* [OP354256]*, Rexea prometheoides* [OP354257]*,* and *Thyrsites atun* [OP598802] calculated in MEGA v11 using concatenated protein-coding genes including stop codons. 3](#_Toc136558140)

[**Table S4**. A subset of partitioning schemes and relevant models used in molecular divergence dating BEAST as predicted by ModelFinder and bModelTest. 4](#_Toc136558141)

[**Figure S2.** Phylogenetic tree showing numbering of internal nodes (see Table S6). 5](#_Toc136558142)

[**Table S6.** 95%HPD values for Figure S2. 6](#_Toc136558143)

# [**Table S1**](Supplementary%20Table%20S1%20-%20Main%20features%20of%20the%20mitochondrial%20genomes%20of%20five%20Gempylidae%20species.xlsx)**.** Main features of the complete mitochondrial genomes of five Gempylidae species; *Neoepinnula orientalis*, *Neoepinnula minetomai*, *Rexea antefurcata*, *Rexea prometheoides,* and *Thyrsites atun*. H-strand–heavy strand, L-strand–light strand; IGN–intergenic regions (+) and overlapping nucleotides (-). n/a. - not applicable. Intergenic spaces were counted manually.

# [**Table S2**](Supplementary%20Table%20S2%20-%20Nucleotide%20Composition%20of%20five%20Gempylidae%20species.xlsx)**.** Nucleotide composition calculated for the major strand for five Gempylidae species.

# **Table S3.** Gene alignment characteristics estimated on MEGA v11, and their corresponding substitution models as predicted by jModelTest.

| **Gene** | **#Nucleotides** | **Conserved** | **Variable** | **Parsim-info** | **Model** |
| --- | --- | --- | --- | --- | --- |
| *ND1* | 972 | 493 | 479 | 432 | TIM3+I+G |
| *ND2* | 1044 | 433 | 611 | 528 | TIM2+I+G |
| *COI* | 1548 | 889 | 659 | 609 | HKY+I+G |
| *COII* | 690 | 335 | 355 | 310 | HKY+I+G |
| *ATPase 8* | 165 | 37 | 128 | 120 | HKY+I+G |
| *ATPase 6* | 681 | 245 | 436 | 402 | TIM2+I+G |
| *COIII* | 783 | 419 | 364 | 334 | HKY+I+G |
| *ND3* | 348 | 160 | 188 | 169 | HKY+I+G |
| *ND4L* | 294 | 122 | 172 | 142 | TrN+I+G |
| *ND4* | 1380 | 601 | 779 | 670 | TIM2+I+G |
| *ND5* | 1836 | 720 | 1116 | 948 | TIM3+I+G |
| *ND6* | 519 | 159 | 360 | 330 | TPM3uf+I+G |
| *Cyt b* | 1140 | 551 | 589 | 540 | HKY+I+G |

# **Figure S1.** Codon usage profile of *Neoepinnula minetomai* [OP359221]*, Neoepinnula orientalis* [OP354258]*, Rexea antefurcata* [OP354256]*, Rexea prometheoides* [OP354257]*,* and *Thyrsites atun* [OP598802] calculated in MEGA v11 using concatenated protein-coding genes including stop codons.

# **Table S4**. A subset of partitioning schemes and relevant models used in molecular divergence dating BEAST as predicted by ModelFinder and bModelTest.

| **Partitioning scheme** | **ModelFinder** | **bModelTest** |
| --- | --- | --- |
| COI_pos3_COII_pos3_ATPase8_pos3_ATPase6_pos3_COIII_pos3_ND4L_pos3 | TIM+F+R3 | 123343 |
| ND1_pos1_COI_pos1_COII_pos1_COIII_pos1_ND3_pos1_ND4L_pos1_CytB_pos1 | TVMe+I+G4 | 121321 |
| ND1_pos2_COI_pos2_COII_pos2_COIII_pos2_ND3_pos2_CytB_pos2 | TVM+F+R2 | 121324 |
| ND1_pos3_ND2_pos3_ND3_pos3_ND4_pos3_ND5_pos3_CytB_pos3 | TIM+F+R3 | 123345 |
| ND6_pos3 | HKY+F+G4 | 121321 |
| ND2_pos1_ATPase8_pos1_ATPase8_pos2_ATPase6_pos1_ND4_pos1_ND5_pos1 | GTR+F+I+G4 | 123453 |
| ND2_pos2_ATPase6_pos2_ND4L_pos2_ND4_pos2_ND5_pos2 | TIM3+F+R3 | 123145 |
| ND6_pos1_ND6_pos2 | TIM3+F+I+G4 | 123143 |

**Table S5.** Estimates of Evolutionary Divergence between *Thyrsites atun* sequences sampled from Chile, Amsterdam and Saint-Paul Islands, Inaccessible Island, and New Zealand. The number of base substitutions per site from between sequences are shown. Analyses were conducted using the Maximum Composite Likelihood model. This analysis involved 5 nucleotide sequences. Codon positions included were 1st+2nd+3rd+Noncoding. All ambiguous positions were removed for each sequence pair (pairwise deletion option). The numbers above the diagonal represent distance between sampling locations in kilometres (in a straight line) and below the diagonal are genetic distances. There was a total of 11400 positions in the final dataset. Evolutionary analyses were conducted in MEGA v11.

|  | South Africa  [OP133162] | Île Saint-Paul  [OP168897] | New-Zealand  [OP598802] | Ina. Island  [OP598801] | Chile  [OP598800] |
| --- | --- | --- | --- | --- | --- |
| South Africa [OP133162] |  | 4,998 | 11,554 | 3,347 | 8,446 |
| Île Saint-Paul [OP168897] | 0,01910 |  | 7,833 | 7,540 | 11,903 |
| New-Zealand  [OP598802] | 0,01983 | 0,01075 |  | 11,278 | 9,131 |
| Ina. Island  [OP598801] | 0,00511 | 0,01966 | 0,02020 |  | 5,175 |
| Chile  [OP598800] | 0,00370 | 0,01993 | 0,02047 | 0,00547 |  |


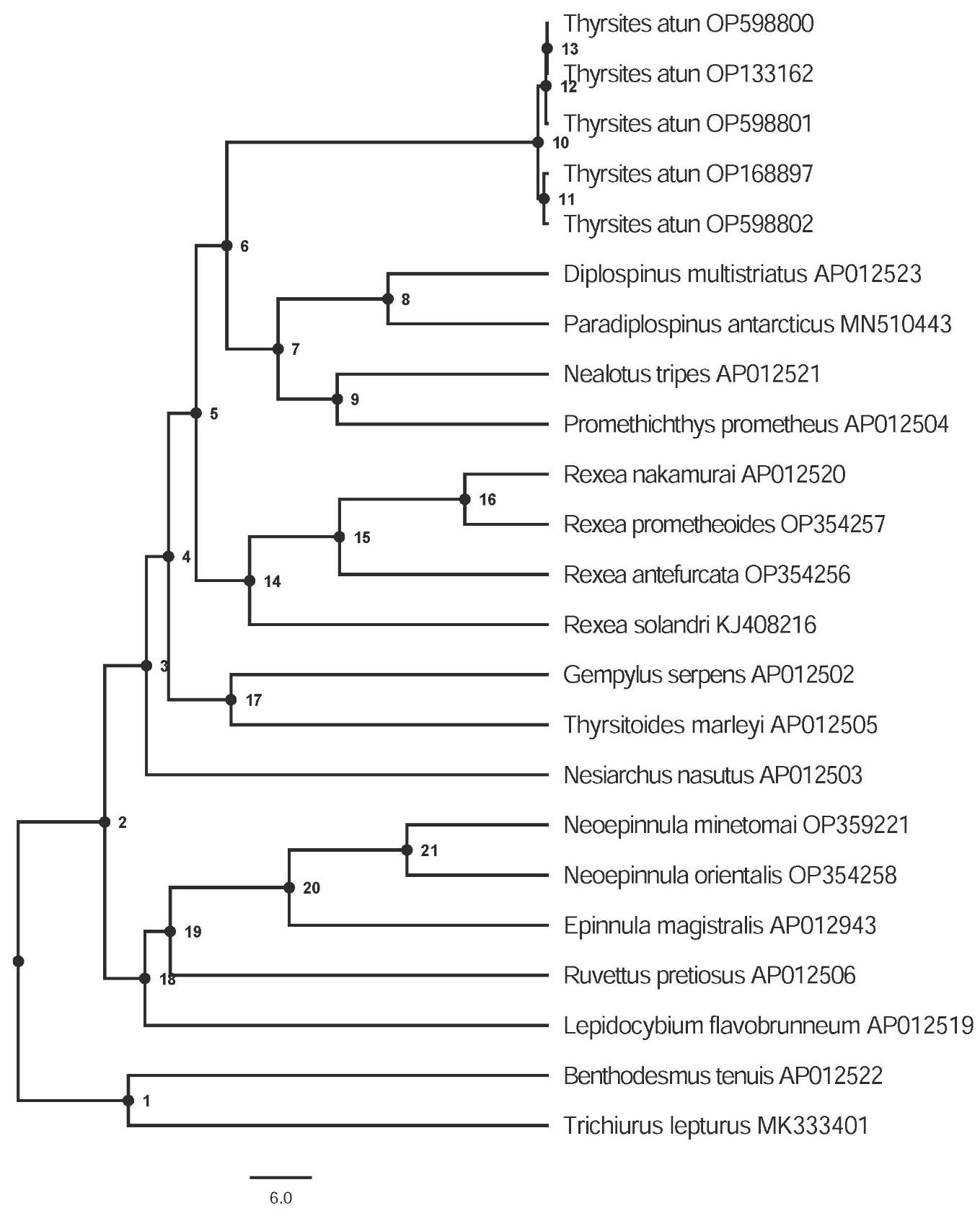


# **Figure S2.** Phylogenetic tree showing numbering of internal nodes (see Table S6).

# **Table S6.** 95%HPD values for Figure S2.

Node ages and 95% HPD (height posterior density) obtained from the BEAST analyses. Node numbers correspond to those presented in Figure S2.

| **Node ID** | **Node ages (My.)** | **95%_HPD[min] (My.)** | **95%_HPD[max] (My.)** |
| --- | --- | --- | --- |
| 1 | 41.0123 | 23.2131 | 59.6161 |
| 2 | 43.3048 | 35.8861 | 52.0391 |
| 3 | 39.2424 | 33.4462 | 46.5406 |
| 4 | 37.0923 | 31.9541 | 43.6940 |
| 5 | 34.4042 | 28.5966 | 41.2440 |
| 6 | 31.4029 | 25.2381 | 38.1842 |
| 7 | 26.4023 | 19.4976 | 32.9028 |
| 8 | 15.6890 | 9.2502 | 22.3497 |
| 9 | 20.6259 | 12.9748 | 27.8605 |
| 10 | 1.0541 | 0.4963 | 1.7694 |
| 11 | 0.4795 | 0.1579 | 0.8741 |
| 12 | 0.2876 | 0.1134 | 0.5093 |
| 13 | 0.1714 | 0.0506 | 0.3215 |
| 14 | 29.1758 | 21.5840 | 36.6072 |
| 15 | 20.4231 | 13.1864 | 27.6953 |
| 16 | 8.1909 | 3.2609 | 13.5515 |
| 17 | 30.9957 | 30.0004 | 31.9546 |
| 18 | 39.4006 | 30.7804 | 48.7046 |
| 19 | 36.9212 | 27.9492 | 46.1889 |
| 20 | 25.3148 | 15.2140 | 35.2213 |
| 21 | 13.8402 | 5.8935 | 22.4557 |
